# Supplementary material for: A Novel Concept of the “Standard Human” in the Assessment of Individual Total Heart Size: Lessons from Non-Contrast-Enhanced Cardiac CT Examinations
Source: Diagnostics (Basel). 2025 Jun 13;15(12):1502. doi: 10.3390/diagnostics15121502 (PMC12192549; doi:10.3390/diagnostics15121502)
Supplement: Supplementary file 1 [file diagnostics-15-01502-s001.zip › diagnostics-3639090-supplementary.pdf]

SUPPLEMENTAL MATERIAL

Table S1. Measured LA, RA, Bi-atrial, and Bi-ventricular volumes (fat-free)

BMI class	Risk	Sex	n	LAV	RAV	BiAV	BIV
0	0	F	285	65.0 ± 15.6	63.4 ± 16.5	128.4 ± 29.8	277.0 ± 53.6
0	0	M	68	74.2 ± 15.4[c]	71.3 ± 17.2[c]	145.5 ± 29.3[c]	361.4 ± 72.4[c]
0	1	F	133	77.2 ± 23.8	70.0 ± 21.2	147.2 ± 41.9	284.2 ± 76.6
0	1	M	93	85.0 ± 26.3[a]	76.5 ± 22.3[a]	161.6 ± 44.0[a]	378.2 ± 83.9[c]
1	0	F	286	72.2 ± 15.2	67.7 ± 14.5	139.9 ± 27.4	287.4 ± 52.7
1	0	M	118	76.7 ± 17.8[a]	75.4 ± 17.4[c]	152.1 ± 33.2[c]	375.3 ± 57.6[c]
1	1	F	289	86.8 ± 26.9	74.2 ± 24.6	161.0 ± 48.2	290.6 ± 64.1
1	1	M	230	102.8 ± 39.7[c]	94.5 ± 34.8[c]	197.3 ± 71.1[c]	430.5 ± 96.5[c]
2	0	F	215	75.6 ± 15.5	69.4 ± 15.5	145.0 ± 28.6	306.6 ± 51.2
2	0	M	49	72.8 ± 15.9	74.9 ± 16.1[a]	147.7 ± 29.6	388.7 ± 57.7[c]
2	1	F	295	97.8 ± 38.6	83.5 ± 31.3	181.3 ± 65.3	332.6 ± 81.5
2	1	M	244	103.3 ± 37.7	97.9 ± 37.3[c]	201.2 ± 71.9[c]	451.7 ± 105.1[c]
All subgroups			2305	84.2 ± 30.6	77.2 ± 27.4	161.4 ± 55.0	338.1 ± 95.7

Abbreviations: BMI category 0 - normal weight, 1 - overweight, 2 - obesity; Risk subgroup 0 - CACS<10, normal blood pressure, non-diabetic, one of smoker and high lipids, Risk group 1 – CACS ≥10 or high BP or t2DM or smoker and high lipids, or structural abnormalities. F – females, M – males, a – p<0.05, c – p<0.001

Table S2. BSA-indexed LA, RA, bi-atrial, and bi-ventricular volumes (fat-free)

BMI class	Risk	Sex	n	LAV	RAV	BiAV	BIV
0	0	F	285	39.5 ± 9.4	38.5 ± 10.1	78.1 ± 18.2	168.2 ± 30.6
0	0	M	68	39.8 ± 7.6	38.3 ± 8.7	78.1 ± 14.5	193.6 ± 33.9 [c]
0	1	F	133	47.0 ± 14.5	42.5 ± 12.2	89.5 ± 24.8	173.0 ± 46.4
0	1	M	93	45.8 ± 14.4	41.1 ± 11.6	86.9 ± 23.6	202.8 ± 41.8 [c]
1	0	F	286	40.3 ± 8.5	37.7 ± 8.1	78.0 ± 15.3	159.9 ± 27.7
1	0	M	118	38.1 ± 9.1 [a]	37.3 ± 8.5	75.4 ± 16.7	185.9 ± 28.9[c]
1	1	F	289	48.6 ± 14.9	41.5 ± 13.2	90.1 ± 26.2	162.7 ± 34.9
1	1	M	230	50.3 ± 18.4	46.2 ± 15.9 [c]	96.3 ± 32.6[a]	210.3 ± 43.0[c]
2	0	F	215	38.1 ± 7.7	34.9 ± 7.5	73.0 ± 14.0	154.3 ± 24.3
2	0	M	49	33.7 ± 7.5[c]	34.7 ± 7.5	68.4 ± 14.0[a]	179.7 ± 25.8[c]
2	1	F	295	49.0 ± 18.8	41.8 ± 15.0	90.9 ± 31.5	166.4 ± 36.1
2	1	M	244	46.2 ± 16.8	43.8 ± 16.8	90.1 ± 32.2	201.6 ± 42.7 [c]
All Groups			2305	44.1 ±14.6	40.4 ± 12.8	84.5 ± 25.7	176.3 ± 39.9

Abbreviations: BMI category 0 - normal weight, 1 - overweight, 2 - obesity; Risk subgroup 0 - CACS<10, normal blood pressure, non-diabetic, one of smoker and high lipids, Risk group 1 – CACS ≥10 or high BP or t2DM or smoker and high lipids, or structural abnormalities. F – females, M – males, a – p<0.05, c – p<0.001

Table S3. Height-indexed LA, RA, Bi-atrial, Bi-ventricular volumes (fat-free)

BMI class	Risk	Sex	n	LAV	RAV	BiAV	BIV
0	0	F	285	40.0 ± 9.4	39.0 ± 10.2	79.0 ± 18.3	170.3 ± 31.5

0	0	M	68	42.5 ± 8.4	40.8 ± 9.5 [a]	83.3 ± 16.0	206.7 ± 37.9 [c]
0	1	F	133	47.7 ± 14.5	43.2 ± 12.7	90.9 ± 25.2	175.7 ± 46.1
0	1	M	93	48.7 ± 15.2	43.7 ± 12.5	92.4 ± 25.1	215.8 ± 45.9 [c]
1	0	F	286	44.6 ± 9.3	41.8 ± 8.9	86.3 ± 16.7	177.2 ± 30.7
1	0	M	118	43.9 ± 10.3	43.1 ± 10.0	87.1 ± 19.2	214.7 ± 33.0[c]
1	1	F	289	53.9 ± 16.5	46.0 ± 14.8	99.9 ± 29.3	180.4 ± 39.2
1	1	M	230	58.3 ± 21.7[b]	53.6 ± 18.8[c]	111.9 ± 38.6[c]	244.3 ± 51.3[c]
2	0	F	215	46.8 ± 9.5	42.9 ± 9.4	89.7 ± 17.5	189.5 ± 30.4
2	0	M	49	42.0 ± 9.3[b]	43.1 ± 9.3	85.1 ± 17.3	223.7 ± 32.3[c]
2	1	F	295	60.8 ± 23.9	51.9 ± 19.1	112.7 ± 40.1	206.6 ± 48.8
2	1	M	244	59.1 ± 21.6	56.0 ± 21.3[a]	115.1 ± 41.1	257.8 ± 57.2[c]
All Groups			2305	50.6 ± 17.8	46.3 ± 15.6	96.8 ± 31.6	202.1 ± 51.0

Abbreviations: BMI category 0 - normal weight, 1 - overweight, 2 - obesity; Risk subgroup 0 - CACS<10, normal blood pressure, non-diabetic, one of smoker and high lipids, Risk group 1 – CACS ≥10 or high BP or t2DM or smoker and high lipids, or structural abnormalities. F – females, M – males, a – p<0.05, c – p<0.001
